# Lemur Tyrosine Kinase 2 (LMTK2) Level Inversely Correlates with Phospho-Tau in Neuropathological Stages of Alzheimer’s Disease

**DOI:** 10.3390/brainsci10020068

**Published:** 2020-01-27

**Authors:** János Bencze, Máté Szarka, Viktor Bencs, Renáta Nóra Szabó, László V. Módis, Dag Aarsland, Tibor Hortobágyi

**Affiliations:** 1 Department of Pathology, Faculty of Medicine, University of Debrecen, 4032 Debrecen, Hungary; 2 MTA-DE Cerebrovascular and Neurodegenerative Research Group, Department of Neurology, University of Debrecen, 4032 Debrecen, Hungary; 3 Horvath Csaba Memorial Institute of Bioanalytical Research, Research Centre for Molecular Medicine, University of Debrecen, 4032 Debrecen, Hungary; 4 Vitrolink Ltd., 4033 Debrecen, Hungary; 5 Institute for Nuclear Research of the Hungarian Academy of Sciences (ATOMKI), 4026 Debrecen, Hungary; 6 Institute of Pathology, Faculty of Medicine, University of Szeged, 6725 Szeged, Hungary; 7 Department of Old Age Psychiatry, Institute of Psychiatry Psychology and Neuroscience, King’s College London, London SE5 8AF, UK; 8 Centre for Age-Related Medicine, SESAM, Stavanger University Hospital, 4011 Stavanger, Norway

**Keywords:** Alzheimer’s disease, LMTK2, neurodegeneration, tau, digital image analysis

## Abstract

Alzheimer’s disease (AD) is the most common neurodegenerative dementia. Mapping the pathomechanism and providing novel therapeutic options have paramount significance. Recent studies have proposed the role of LMTK2 in AD. However, its expression pattern and association with the pathognomonic neurofibrillary tangles (NFTs) in different brain regions and neuropathological stages of AD is not clear. We performed chromogenic (CHR) LMTK2 and fluorescent phospho-tau/LMTK2 double-labelling (FDL) immunohistochemistry (IHC) on 10–10 postmortem middle frontal gyrus (MFG) and anterior hippocampus (aHPC) samples with early and late neuropathological Braak tau stages of AD. MFG in early stage was our ‘endogenous control’ region as it is not affected by NFTs. Semiquantitative CHR-IHC intensity scoring revealed significantly higher (*p* < 0.001) LMTK2 values in this group compared to NFT-affected regions. FDL-IHC demonstrated LMTK2 predominance in the endogenous control region, while phospho-tau overburden and decreased LMTK2 immunolabelling were detected in NFT-affected groups (aHPC in early and both regions in late stage). Spearman’s correlation coefficient showed strong negative correlation between phospho-tau/LMTK2 signals within each group. According to our results, LMTK2 expression is inversely proportionate to the extent of NFT pathology, and decreased LMTK2 level is not a general feature in AD brain, rather it is characteristic of the NFT-affected regions.

## 1. Introduction

Alzheimer’s disease (AD) is the most common cause of dementia, affecting more than 47 million people worldwide [1]. It was first described by Alois Alzheimer in 1906 [2]. A century later AD is one of the most important public health issues of aging societies. According to our current knowledge, the disease is incurable. Therefore, researchers spare no effort to identify potential therapeutic targets. However, this is not possible without mapping the pathological processes leading to AD. The major neuropathological changes are the deposition of extracellular β-amyloid plaques and intracellular neurofibrillary tangles (NFTs). The main constituent of NFTs is the microtubule-associated protein tau. It is involved in myriad of physiological cellular functions such as stabilization of microtubules, axonal transport, synaptic transmission or activation of unfolded protein response [3,4,5,6]. Under pathological circumstances, the phosphorylation level of tau is 3- to 4-fold higher than in normal brains [7]. Hyperphosphorylated tau is prone to self-aggregation as well as to gain toxic ability through sequestering normal tau and other microtubule-associated proteins, resulting in microtubule disassembly [8]. Braak and Braak first described the hierarchy of NFT spreading in the central nervous system [9]. They established six stages based on the affected brain regions: transentorhinal stages (I-II); limbic stages (III-IV); and isocortical stages (V-VI). In general, NFT burden inversely correlates with the number of surviving neurons, proposing that neurofibrillary lesions have a key role in degenerative changes and apoptotic cell loss in AD [10]. The latest studies suggest that there is a link between the expression/activity of recently described lemur tyrosine kinase 2 (LMTK2) protein and tau hyperphosphorylation [11,12,13]. Despite its name, LMTK2 is a member of a membrane-anchored serine/threonine-specific protein kinase family [14,15,16]. The enzyme is involved in the orchestration of vital physiological processes such as axonal transport, apoptosis or neurogenesis [11,12,14,17,18]. Alterations of LMTK2 level may contribute to neurodegeneration by disrupted axonal transport, enhanced apoptosis and tau hyperphosphorylation [11]. We previously described decreased LMTK2 level in human postmortem AD brains compared to age-matched controls [13]. Our immunohistochemical (IHC) results were in accordance with other human and animal model studies [12,19]. However, the expression pattern of LMTK2 in different brain regions and neuropathological stages of AD has not been investigated yet. Moreover, the question as to whether LMTK2 reduction is a general feature in AD brains or linked to the distribution of NFT pathology remains to be answered. In this study we evaluate the immunolabelling intensity score of the protein, as well as the percentage distribution of phospho-tau/LMTK2 signals in different brain regions and neuropathological (Braak and Braak) stages of AD.

## 2. Materials and Methods

### 2.1. Patients and Samples

The archived tissue samples were drawn from a clinic-pathologic cohort study, the Dementia Study in Western Norway (DemVest) from the Centre for Age-Related Medicine, Stavanger University Hospital, Stavanger, Norway. Selection of cases was based on the neuropathological Braak tau staging. We obtained formalin-fixed paraffin-embedded samples of patients (*n* = 10 in total) with early (Braak stage III or less, *n* = 5) and late stage (Braak stage VI, *n* = 5) pathological changes (Table 1). The majority of the patients in the early neuropathological stage group had mild dementia. In the late neuropathological stage group, every patient suffered from severe dementia. Participants were included at time of diagnosis of dementia and followed annually until death. Dementia was diagnosed according to DSM IV criteria, and AD was diagnosed according to the National Institute of Neurological and Communicative Disorders and Association. Mild dementia was defined as mini-mental state examination (MMSE) score ≥ 20 and/or Clinical Dementia Rating score = 1. The clinical evaluation included standardized scales, and cognition was measured using MMSE and a neuropsychological test battery. In addition, blood tests and MRI scans were performed to rule out other causes for cognitive decline. More details of the study design are provided in our previous work [20]. Block taking for histological and immunohistochemical studies and neuropathological assessment for neurodegenerative diseases was carried out in accordance with standard criteria as described in detail in earlier studies [21].

We selected two brain regions based on the characteristic distribution of NFTs in these stages: (a) anterior hippocampus (aHPC) affected in early neuropathological stages, and (b) middle frontal gyrus (MFG) showing significant pathological changes in the late neuropathological Braak tau stages of AD. Thus, we established four experimental groups. In this setting, MFG in early neuropathological stage served as an endogenous control region because it is spared from NFTs, whereas the other regions (MFG in late stage and aHPC in both stages) are severely affected by NFT pathology.

All participants signed informed consent to participate in the study and to autopsy. The study was approved by the regional committee for medical and health research ethics in Western Norway (REK 2010/633); the Hungarian Medical Research Council, Scientific and Research Ethics Board (19312/2016/EKU); Institutional Ethics Committee of the MRC London Neurodegenerative Diseases Brain Bank (18/WA/0206) at the Institute of Psychiatry Psychology and Neuroscience, King’s College London.

### 2.2. CHR-IHC

The applied immunohistochemical procedure was optimized in our previous work [13]. After routine dewaxing, sections were treated in 3% (v/v) H_2_O_2_:methanol solution for 30 min to block endogenous peroxidase enzymes. Heat-induced epitope retrievals were carried out in a microwave oven (5 min at 800 W, 2 × 5 min at 250 W). Non-specific sites were blocked with 10% (v/v) normal goat serum for an hour at room temperature. Sections were incubated with anti-KPI-2 (LMTK2) primary antibodies (clone H9, SantaCruz Biotechnology) in 1:100 concentration overnight at 4 °C. Goat anti-mouse biotinylated secondary antibody (Agilent/Dako) were applied in 1:200 concentration for an hour at room temperature. For visualization, VECTASTAIN Elite ABC HRP Kit (Vector laboratories) and 3,3’-diaminobenzidine tetrahydrochloride (DAB) reagent (Sigma-Aldrich 10 mg tablets) were used. Nuclear counterstain, antigen retrieval solution and working solution (rinsing and dilution) were Harris hematoxylin, Tris(hydroxymethyl)aminomethane-Ethylenediaminetetraacetic acid (TRIS-EDTA; 10 mM TRIS Base, 1 mM EDTA, pH 9.0) and 20 mmol TRIS-buffered saline (Sigma-Aldrich, TRIS-Buffered Saline 20× solution, 20 mmol Tris, pH 7.4), respectively.

### 2.3. FDL-IHC

Dewaxing, antigen retrieval, nonspecific site blocking and preparation of primary anti-LMTK2 antibody were the same as with the CHR-IHC method described above. However, in FDL-IHC endogenous peroxidase blocking, biotinylated secondary antibody, chromogenic reagents and hematoxylin counterstain are not applicable. Besides anti-LMTK2, anti-phospho-tau (Ser202, Thr205) monoclonal primary antibody (clone AT8, Thermo Fisher Scientific) was also used. Concentrations were 1:100 and 1:500 for anti-LMTK2 and anti-phospho-tau antibodies, respectively. Considering that both primary antibodies are produced in mice, we applied fluorophore-conjugated fragment antigen-binding (Fab) anti-fragment crystallizable (Fc)-region secondary antibodies (FabuLight, Jackson ImmunoResearch Europe Ltd.). Two different fluorophore-conjugated antibodies (Alexa Fluor 488 and Alexa Fluor 594—AffiniPure Fab Fragment Goat Anti-Mouse IgG) were used. These secondary antibodies bind directly to the Fc-region of primary antibodies and spare the antigen-specific Fab region, therefore were incubated separately in vitro with the anti-LMTK2 and anti-phospho-tau primary antibodies. Next, we applied these ‘sandwiches’ to the sections and incubated them overnight at 4 °C. To avoid autofluorescence, samples were treated with Vector TrueVIEW Autofluorescence Quenching Kit (Vector laboratories). Vectashield Antifade Mounting Medium (Vector laboratories) was used for coverslipping. Working solution was 10× pH 7.3 phosphate-buffered saline (Biocare Medical, PBS Plus).

### 2.4. Semiquantitative Analysis for CHR-IHC

**Figure 1:**
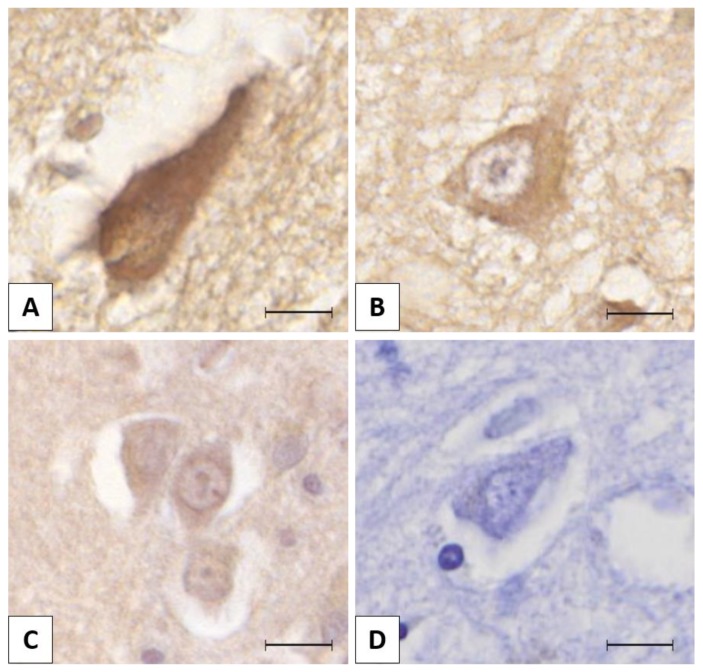
The figure presents different lemur tyrosine kinase 2 (LMTK2) immunolabelling intensities in neurons: (**A**) strong positivity (3+); (**B**) moderate positivity (2+); (**C**) mild positivity (1+); (**D**) negative (0). The protein was visualized by 3,3′-diaminobenzidine (DAB) chromogen. Nuclear counterstain with hematoxylin. Scale bar: 10 µm.

Slides were scanned with Panoramic MIDI II (3DHISTECH Ltd.). Using Panoramic Viewer software (3DHISTECH Ltd.), we took 10-10 high magnification (400×) random consecutive images per sample. The region of interest (ROI) regarding the localization of LMTK2 and NFTs was the transentorhinal region and the neocortex in aHPC and MFG, respectively. Semiquantitative intensity scoring was performed with ImageJ software. Using Cell Counter module, we marked and counted the differentially labelled neurons (0, +1, +2, +3) on every image (Figure 1). In the two key neuroanatomical regions (MFG and aHPC) 1215 neurons were assessed in different neuropathological Braak tau stages of AD. There were 538 (1+) and 418 (2+) cells in the four experimental groups. The strongest (3+) immunolabelling was observed exclusively in the early neuropathological stage MFG group (177 cells). We determined the average labelling intensity scores for each case. Then mean values for aHPC and MFG groups in both neuropathological stages were also calculated.

### 2.5. FDL-IHC Image Analysis

Immunofluorescence microscopy was performed with an Axio Imager Z2 microscope equipped with 20×/0.5 420350-9900 EC Plan-Neofluar objective, Coolcube 1m S/N: 003274. 60N-C 1" 1.0× 426114 camera and appropriate filter sets (Carl Zeiss AG). For digital image acquisition we used Isis fluorescence imaging platform (MetaSystems Hard & Software GmbH). Five photos/cases were taken randomly at medium magnification (200×) with the same illumination intensities, exposure times and camera settings.

For digital image analysis of phospho-tau (green) and LMTK2 (red) fluorescent signals ImageJ software was used applying the following subtle methods: (a) Merged (red and green channels superimposed) images belonging to the same case were gathered into a stack. RGB stack images were downgraded to 8-bit grey intensity scale, but the original red and green channel data were kept for redirected measurements, in order to be able to perform quantitative analysis on the two proteins. (b) Thresholding was executed with values manually adjusted if needed. (c) For neuronal phospho-tau/LMTK2 signal quantification, objects greater than 400 pixel size were processed with the aforementioned red and green channel redirection type measurements, resulting in red and green channel raw integrated density data columns corresponding to the same area value of the given ROI on the image.

Based on the relation of phospho-tau and LMTK2, our assumption was that the sum of their signals can be considered invariable on the fluorescent images. Direct comparison of their relative intensity on the images was performed by evaluating the proportion of the green (phospho-tau) and red (LMTK2) signals, respectively, against their sum in every case. We analyzed more than fourteen thousand ROIs altogether. After the case-based evaluation, group level analysis was performed by transforming the red and green channel data.

Transformation was based on the following equation:

R − G/R + G = X(1)

where R is the percentage of measured red (LMTK2) signal, G is the percentage of measured green (phospho-tau) signal and X can take a value between +1 and −1. If X = +1, this means that 100% red signal was measured, and if X = −1, then 100% green signal was observed, corresponding to the measured neurons of groups.

It is important to emphasize that while CHR-IHC analysis was carried out to compare the mean LMTK2 intensity scores between the groups, FDL-IHC was performed in order to evaluate the percentage distribution of phospho-tau/LMTK2 signals within an experimental group.

### 2.6. Statistical Analysis

**Figure 2:**
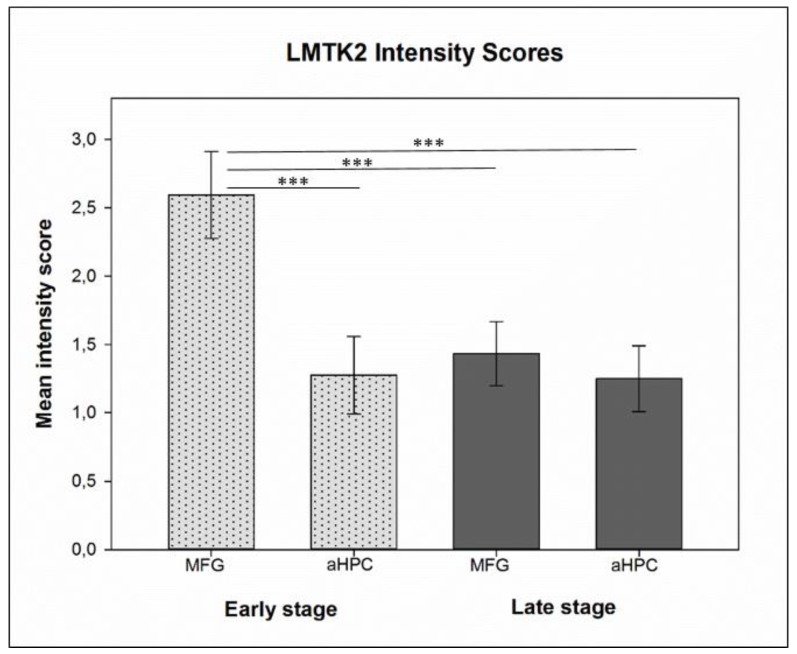
Histogram depicts the mean chromogenic lemur tyrosine kinase 2 (LMTK2) intensity scores in the middle frontal gyrus (MFG) and anterior hippocampus (aHPC) brain regions in early (dotted light gray columns) and late (dark gray columns) neuropathological Braak tau stage cases. Here, *t*-test showed statistically significant (*p* < 0.001 (***)) differences between pairwise comparison of the mean intensity scores of early neuropathological stage MFG group (endogenous control—spared from neurofibrillary tangles (NFTs)) vs. NFT-affected groups (aHPC in early neuropathological stage and both regions in late neuropathological stage).

In the case of CHR-IHC both normality test (Shapiro-Wilk) and equal variance test passed, allowing us to apply *t*-test to compare pairwise the mean intensity scores of the groups: early stage MFG vs. early stage aHPC, early stage MFG vs. late stage MFG, early stage MFG vs. late stage aHPC, late stage MFG vs. early stage aHPC, late stage MFG vs. late stage aHPC, early stage aHPC vs. late stage aHPC (Figure 2). In addition, analysis of covariance (ANCOVA) was also run to test to influence of age, final MMSE score and APOE gene polymorphism on the LMTK2 results. We used SPSS 25 (IBM Corp.) software for statistical analysis; the dependent variable was LMTK2 CHR-IHC data, fixed factors were Braak tau stages and the brain regions, while covariates were age, final MMSE score and APOE gene polymorphism. FDL-IHC data did not follow normal distribution (Shapiro-Wilk test failed), therefore Spearman’s correlation test was applied to define the association between phospho-tau and LMTK2 fluorescent signals within the experimental groups (Table 2).

## 3. Results

### 3.1. Chromogenic Immunohistochemistry (CHR-IHC)

We assessed 1215 neurons across different neuropathological Braak tau stages of AD in the two key brain regions (MFG and aHPC). The average labelling intensity of cases varied between 2.13–2.92, 1.07–1.7, 1.22–1.81 and 1.00–1.55 in the early neuropathological stage MFG, early neuropathological stage aHPC, late neuropathological stage MFG and late neuropathological stage aHPC, respectively. However, their standard deviations were almost identical and results followed normal distribution rendering them suitable for statistical analysis. The calculated mean intensity scores were 2.59, 1.28, 1.43 and 1.24 for early neuropathological stage MFG, aHPC and late neuropathological stage MFG, aHPC, respectively.

In the three NFT-affected brain regions (aHPC in early neuropathological stage and aHPC and MFG in late neuropathological stage) we detected statistically significant alteration (*p* < 0.001) in the mean LMTK2 immunolabelling intensity scores compared to the relatively spared middle frontal gyrus in early neuropathological stage (Figure 2). Among the LMTK2 intensity scores of the three NFT-affected regions there were no statistically significant differences. According to ANCOVA, neither age (*p* = 0.137) nor final MMSE score (*p* = 0.132) nor APOE gene polymorphism (*p* = 0.253) significantly influenced the LMTK2 CHR-IHC results.

### 3.2. Fluorescent Double-Labelling Immunohistochemistry (FDL-IHC)

**Figure 3:**
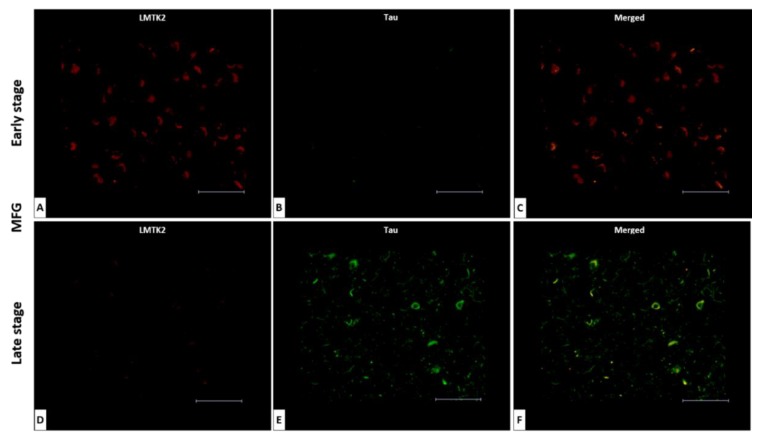
Lemur tyrosine kinase 2 (LMTK2) and phospho-tau fluorescent double-labelling immunohistochemistry in the middle frontal gyrus (MFG) in early (**A**–**C**) and late (**D**–**F**) neuropathological Braak tau stages. LMTK2 immunolabelling (red) dominates the early neuropathological stage (**A,C**), which is spared by neurofibrillary tangles (NFT), while there is an obvious phospho-tau burden (**E,F**) with decreased LMTK2 positivity (**D**) in the late neuropathological stage. LMTK2 and phospho-tau were visualized by Alexa Fluor 594 and Alexa Fluor 488 fluorescent dyes, respectively. Scale bar: 50 µm.

**Figure 4:**
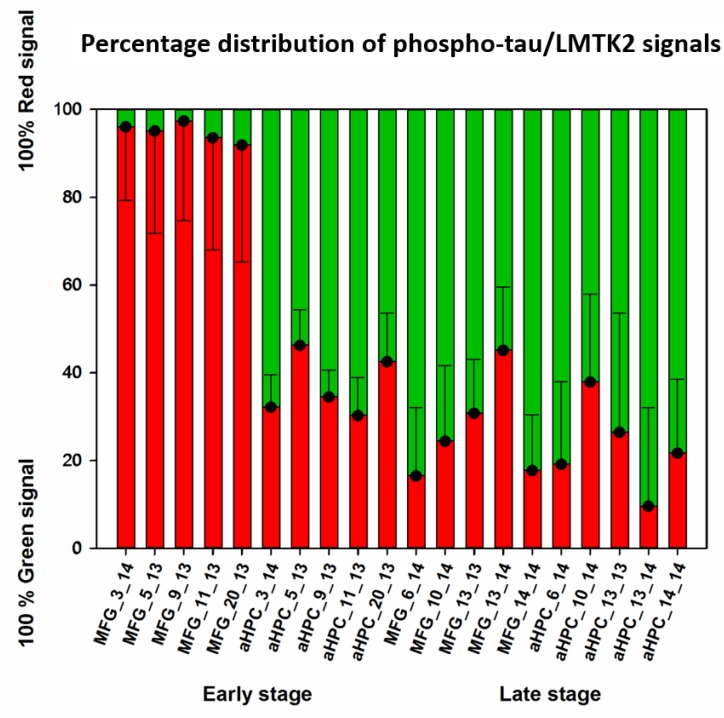
Bars depict the mean level (in %) of fluorescence for red (lemur tyrosine kinase-2 (LMTK2)) and green (phospho-tau) channels of images from the middle frontal gyrus (MFG) and anterior hippocampus (aHPC) in early and late neuropathological Braak tau stages.

**Figure 5:**
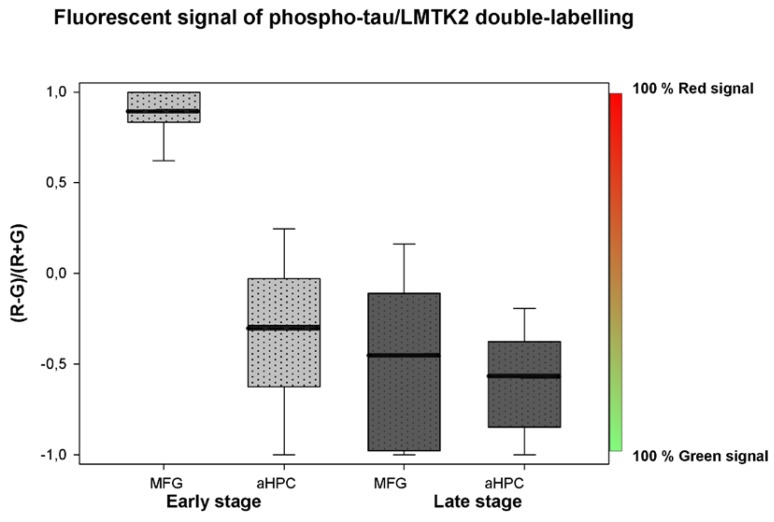
Phospho-tau and lemur tyrosine kinase 2 (LMTK2) double-labelling fluorescent immunohistochemistry signals of the middle frontal gyrus (MFG) and anterior hippocampus (aHPC) in early (dotted light gray boxes) and late (dotted dark gray boxes) neuropathological Braak tau stages are quantified. Boxes represent the interquartile range of the fluorescence signal of phospho-tau (green) and LMTK2 (red) on a unified scale (−1 to +1) with mean levels indicated by black lines. (−1 corresponds to 100% phospho-tau signal compared to 0% LMTK2; +1 means 100% LMTK2 signal and 0% phospho-tau.).

Phospho-tau/LMTK2 FDL-IHC showed LMTK2 predominance in the endogenous control group (MFG in early neuropathological stage), while phospho-tau overburden and decreased LMTK2 immunolabelling were detected in NFT-affected groups (aHPC in early and both regions in late neuropathological stage) (Figure 3). The measured percentage distribution of phospho-tau/LMTK2 values of the individual cases are visualized in Figure 4. Group level comparison of LMTK2 (red) and phospho-tau (green) fluorescent signals, derived from the case-based evaluation, are shown in Figure 5.

Statistical analysis (Spearman’s correlation) was performed on the red (LMTK2) and green (phospho-tau) channel data of more than fourteen thousand ROIs to assess correlation. Perfect negative correlation (Spearman rank order correlation coefficient = −1) was observed in each experimental group. Statistical information for early and late neuropathological stage groups is shown in Table 2.

## 4. Discussion

The CHR-IHC immunolabelling of LMKT2 is significantly decreased in the NFT-affected regions compared to the relatively spared endogenous control region. Visualization of NFTs with phospho-tau/LMTK2 FDL-IHC further supported the CHR-IHC findings. In the endogenous control group LMTK2 immunolabelling dominated over the sparse phospho-tau positivity, while the NFT-affected regions displayed decreased LMTK2 immunopositivity (Figure 3). Pearson’s statistical test showed perfect negative correlation with phospho-tau/LMTK2 FDL signals within each group. This means that the expression of LMTK2 is inversely proportionate to the extent of phospho-tau/NFT pathology. ANCOVA further strongly supported these findings, because neither age nor final MMSE nor APOE gene polymorphism significantly influenced the LMTK2 CHR-IHC results. Consequently, LMTK2 reduction is not a general feature of AD brains, rather it is characteristic of the NFT-affected regions.

In accordance with our findings, recent studies have shown decreased LMTK2 level in AD [12,13,19]. A genome-wide gene expression analysis detected reduced LMTK2 expression in the cortex of transgenic Tau P301L mice [19]. Mórotz et al. recently reported a western blot analysis on postmortem human brain tissues [12]. They established three experimental groups based on the Braak tau stages (control—Braak I–II; mild dementia—Braak III–IV; severe dementia—Braak V–VI). The expression of LMTK2 decreased in the cortex with the progression of NFT pathology. However, the investigated region(s) of the frontal cortex were not specified, thus direct comparison with our results is not possible. In our previous paper we reported significantly decreased immunolabelling in the middle frontal gyrus of patients with Braak stage VI pathology compared to age-matched controls and neocortical Lewy body disease (LBD) cases [13]. Because LBD-specific Lewy body-bearing neurons showed strong LMTK2 immunoreaction and Lewy bodies themselves were negative, we hypothesized that the decreased average immunopositivity (compared to controls) was due to coexisting AD-type pathology. The association between LMTK2 expression and the regional distribution of NFT pathology has been confirmed experimentally in the current study. However, the question as to whether LMTK2 reduction or tau hyperphosphorylation is the primary event remains to be answered.

In a recent review on LMTK2 we proposed that decreased protein activity or expression may contribute to tau hyperphosphorylation [11]. While analysis of LMTK2 signaling is beyond the scope of our current work, recent cell biological studies have shed light on the interaction between phospho-tau and LMTK2. Under physiological circumstances, LMTK2 is activated by cyclin-dependent kinase 5 (CDK5)/p35-mediated phosphorylation [22], hence LMTK2 phosphorylates the catalytic subunit of protein phosphatase 1 (PP1) leading to the inhibition of the enzyme [17,22,23]. PP1 cannot effectively remove inhibitory phosphoryl groups from glycogen synthase kinase-3β (GSK3β), resulting in decreased GSK3β activity [17,23,24]. CDK5 and GSK3β are well-known major tau kinases [25,26,27,28]. Physiologically, CKD5 can inhibit GSK3β indirectly via the LMTK2-mediated signaling pathway [29,30,31], whereas in AD, caspase overactivation leads to cleavage of cofactor p35 into p25 and p10 [32,33,34,35,36,37]. The generated CDK5/p25 complex has prolonged half-life and activity contributing to tau hyperphosphorylation [33,36,37,38]. Interestingly, p25 exhibited weaker binding affinity to LMTK2 than p35 in yeast two-hybrid screen [22] suggesting that prolonged CDK5/p25 activity induces less LMTK2 activation than CDK5/p35. The decreased LMTK2 activity results in the disinhibition of PP1 as well as the indirect overactivation of GSK3β, which is a critical event in AD pathogenesis [25,26,28].

In AD the functional impairment of LMTK2 is associated with its decreased expression. However, there are no available experimental data on the mechanism(s) leading to reduced LMTK2 level. Nonetheless, a recent theory suggesting that neurodegeneration is the cancer of neurons may serve as a potential explanation [39,40,41]. Because neurons are terminally differentiated cells it is possible that certain noxious stimuli which cause uncontrolled proliferation in other organs result in aberrant cell cycle re-entry with degenerative changes and apoptosis in nerve cells [42,43,44,45,46,47]. The role of LMTK2 has been investigated in several malignancies, particularly in prostate cancer [48,49,50,51]. Harries et al. reported reduced LMTK2 expression in prostate adenocarcinoma, compared to benign prostate hyperplasia [50]. They proposed that a previously described [52] rs6465657 single nucleotide polymorphism (SNP) in the intron 9 of LMTK2 in prostate cancer is a loss-of function variant contributing to tumorigenesis; thereby, LMTK2 acts as a tumor suppressor/proapoptotic protein. Aberrant cell cycle reactivation increases cyclin D and cyclin E expression as well as enhances the G_1_ and G_2_ phase cyclin dependent kinases’ (CDKs) activity resulting in retinoblastoma protein phosphorylation and accompanying E2F release in neurons [53,54,55,56,57,58]. E2F suppresses the expression of proapoptotic genes and transactivates downstream cell cycle genes, leading to the progression of this ultimately lethal cycle [59]. Theoretically, considering LMTK2 as a proapoptotic protein, it is possible that E2F reduces its level by transcriptional silencing. However, the interaction between E2F and the LMTK2 gene remains to be proven.

## 5. Conclusions

In summary, there is a negative correlation between the regional expression of LMTK2 and the severity of NFT pathology, validated by both CHR-IHC and FDL-IHC methods. While CHR-IHC analysis compared the mean LMTK2 intensity scores based on Braak tau staging, FDL-IHC evaluated the phospho-tau/LMTK2 percentage distribution within each experimental group. Age, final MMSE and APOE gene polymorphism did not influence the LMTK2 CHR-IHC results. It has been demonstrated that LMTK2 reduction is strongly associated with NFT pathology and it is not a tau-independent feature. Further studies are needed to explore the pharmacological manipulation of LMTK2-related pathways [60] as a therapeutic option in AD.

## Figures and Tables

**Table 1:** Human postmortem samples: case identifier (study ID), age (baseline), sex, final MMSE score, neuropathological Braak tau stage and APOE gene polymorphism. (M: male; F: female; MMSE: mini-mental state examination; APOE: apolipoprotein E).

Study ID	Sex	Age (Baseline)	Final MMSE	Braak Tau Stage	APOE Gene Polymorphism
HT-05-13	M	70	25	II	ε3–ε3
HT-09-13	M	77	22	III	ε3–ε3
HT-11-13	F	55	23	I	ε3–ε4
HT-20-13	M	64	26	I	-
HT-03-14	M	72	16	III	ε3–ε3
HT-13-13	F	80	13	VI	ε3–ε4
HT-14-13	F	84	13	VI	ε3–ε3
HT-06-14	M	77	6	VI	ε3–ε4
HT-10-14	M	68	7	VI	ε4–ε4
HT-14-14	M	55	0	VI	ε3–ε3

**Table 2:** Statistical analysis of lemur tyrosine kinase 2 (LMTK2) (red)/phospho-tau (green) fluorescent signal correlation in the middle frontal gyrus (MFG) and anterior hippocampus (aHPC) in early and late neuropathological Braak tau stages

	Early stage				Late stage			
	MFG		aHPC		MFG		aHPC	
**Spearman Rank Order Correlation Coefficient**	−1		−1		−1		−1	
***p*-Value**	0.0000002		0.0000002		0.0000002		0.0000002	
**Mean (Red; Green)**	0.946	0.054	0.349	0.651	0.273	0.727	0.215	0.785
**Standard deviation**	0.087		0.233		0.237		0.159	
**Standard error**	0.00175		0.00468		0.00349		0.00238	
**Number of pairs examined**	2464		2478		4609		4457	

## Abbreviations

ANCOVA	Analysis of covariance
AD	Alzheimer’s disease
APOE	Apolipoprotein E
aHPC	Anterior hippocampus
CDK5	Cyclin-dependent kinase 5
CHR	Chromogenic
DAB	3,3’-diaminobenzidine tetrahydrochloride
EDTA	Ethylenediaminetetraacetic acid
Fab	Fragment antigen-binding
Fc	Fragment crystallizable
FDL	Fluorescent double-labelling
GSK3β	Glycogen synthase kinase-3β
IHC	Immunohistochemistry
LBD	Lewy body disease
LMTK2	Lemur tyrosine kinase 2
MFG	Middle frontal gyrus
MMSE	Mini-mental state examination
NFT	Neurofibrillary tangles
PP1	Protein phosphatase 1
ROI	Region of interest
SNP	Single nucleotide polymorphism
